# Do as AI say: susceptibility in deployment of clinical decision-aids

**DOI:** 10.1038/s41746-021-00385-9

**Published:** 2021-02-19

**Authors:** Susanne Gaube, Harini Suresh, Martina Raue, Alexander Merritt, Seth J. Berkowitz, Eva Lermer, Joseph F. Coughlin, John V. Guttag, Errol Colak, Marzyeh Ghassemi

**Affiliations:** 1 Department of Psychology, University of Regensburg, Regensburg, Germany; 2 MIT AgeLab, Massachusetts Institute of Technology, Cambridge, MA USA; 3 MIT Computer Science & Artificial Intelligence Lab, Massachusetts Institute of Technology, Cambridge, MA USA; 4 Boston Medical Center, Boston, MA USA; 5 Department of Radiology, Beth Israel Deaconess Medical Center, Boston, MA USA; 6 LMU Center for Leadership and People Management, LMU Munich, Munich, Germany; 7 FOM University of Applied Sciences for Economics & Management, Munich, Germany; 8 Li Ka Shing Knowledge Institute, St. Michael’s Hospital, Toronto, Canada; 9 Department of Medical Imaging, University of Toronto, Toronto, Canada; 10 Departments of Computer Science and Medicine, University of Toronto, Toronto, Canada; 11 Vector Institute, Toronto, Canada

**Keywords:** Radiography, Human behaviour, Decision making

## Abstract

Artificial intelligence (AI) models for decision support have been developed for clinical settings such as radiology, but little work evaluates the potential impact of such systems. In this study, physicians received chest X-rays and diagnostic advice, some of which was inaccurate, and were asked to evaluate advice quality and make diagnoses. All advice was generated by human experts, but some was labeled as coming from an AI system. As a group, radiologists rated advice as lower quality when it appeared to come from an AI system; physicians with less task-expertise did not. Diagnostic accuracy was significantly worse when participants received inaccurate advice, regardless of the purported source. This work raises important considerations for how advice, AI and non-AI, should be deployed in clinical environments.

## Introduction

The data-intensive nature of healthcare makes it one of the most promising fields for the application of artificial intelligence (AI) and machine learning algorithms^1–3^. Applications of AI in classifying medical images have demonstrated excellent performance in several tasks, often on par with, or even above, that of human experts^4,5^. However, it is not clear how to effectively integrate AI tools with human decision-makers; indeed, the few cases where systems have been implemented and studied showed no improved clinical outcomes^6,7^.

AI systems will only be able to provide real clinical benefit if the physicians using them are able to balance trust and skepticism. If physicians do not trust the technology, they will not use it, but blind trust in the technology can lead to medical errors^8–11^. The interaction between AI-based clinical decision-support systems and their users is poorly understood, and studies in other domains have garnered inconsistent and complex findings. Reported behaviors include both a skepticism or distrust of algorithmic advice (*algorithmic aversion*)^12–14^ and more willingness to adhere to algorithmic advice over human advice (*algorithmic appreciation*)^15,16^. Responses can vary depending on the task at hand or the person’s task expertise*—*for instance, one study found that algorithmic appreciation waned when the participants had high domain expertise^15,17^. It is therefore important to study how physicians of different expertise levels will perceive and integrate AI-generated advice before such systems are deployed^18–20^.

One proposed study framework is to measure clinical task performance with and without AI assistance^10^. In practice, physicians have the ability to solicit opinions from other practitioners^21^, and can be aided by clinical decision-support systems^22^. Comparing AI advice to human advice, then, allows us to more directly study the situations in which physicians are faced with advice before having the opportunity to make their own judgment. Moreover, verifying or disagreeing with given advice is a different task than generating an answer from scratch. Considering two conditions where advice is consistently received but the source is varied allows for a more direct comparison. In this work, we evaluated the impact of advice from different purported sources on a specific clinical task. Because AI-enabled diagnostic technology has made significant advances in radiology^23,24^, we recruited physicians with different levels of task expertise to perform a radiological task. We created inaccurate and accurate clinical advice, determined by human experts. We then artificially reported this advice as coming from an AI-based system or experienced human radiologist. We assessed whether the purported source of diagnostic advice (AI or human) influenced two dependent variables: (1) perception of advice quality and (2) participants’ diagnostic accuracy. We also investigated whether the accuracy of the advice given to the participants had an effect on the two dependent variables.

## Results

### Experiment

The participants were physicians with different levels of task expertise: radiologists (*n* = 138) were the high-expertise group and physicians trained in internal/emergency medicine (IM/EM, *n* = 127) were the lower expertise group (because they often review chest X-rays, but have less experience and training than radiologists).

**Fig. 1:**
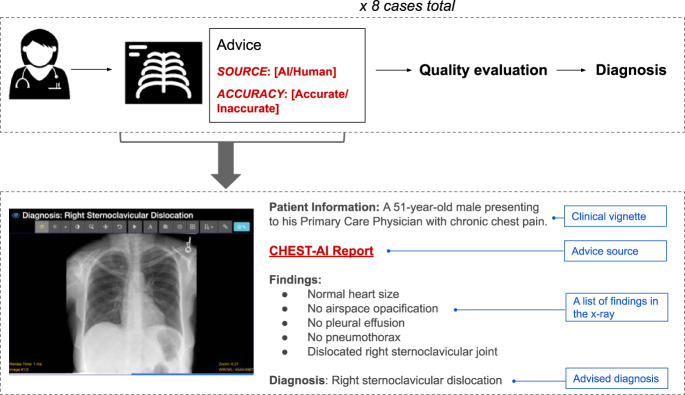
Overview of the experiment. Each participant reviewed eight cases. For each case, the physician would see the chest X-ray as well as diagnostic advice, which would either be accurate or inaccurate. The advice was labeled as coming either from an AI system or an experienced radiologist. Participants were then asked to rate the quality of the advice and make a final diagnosis.

We selected eight cases, each with a chest X-ray, from the open source MIMIC Chest X-ray database^20^. Participants were provided with the chest X-rays, a short clinical vignette, and diagnostic advice that could be used for their final decisions. They were asked to (1) evaluate the quality of the advice through a series of questions, and (2) make a final diagnosis (see Fig. 1).

The experiment followed a 2 × 2 mixed factorial design to test the impact of the source of the advice (AI vs. human) as the between-subject factor, and accuracy of the advice (accurate vs. inaccurate) as the within-subject factor. Participants were randomly assigned six cases with accurate advice and two cases with inaccurate advice. Two mixed-effects models were calculated for the two dependent variables: (1) quality rating of the advice (derived from responses to multiple questions) and (2) accuracy of the final diagnoses. Both dependent variables were regressed on the accuracy of the advice (accurate vs. inaccurate), the source of the advice (AI vs. human), and the interaction between accuracy and source. We also controlled for several individual-related variables.

### Advice quality ratings display algorithmic aversion in experts

**Fig. 2:**
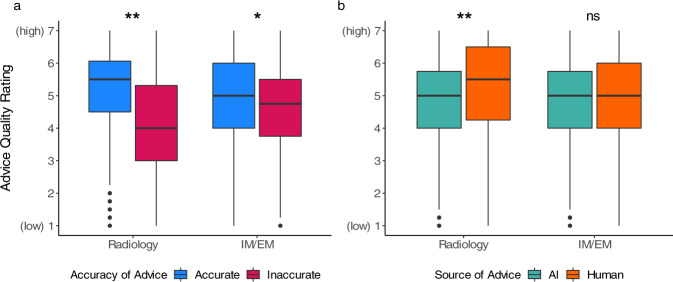
Advice quality rating across advice accuracy and source. We demonstrate the effect of the accuracy of advice and source of advice on the quality rating across both types of physicians: task experts (radiologists), and non-experts (IM/EM physicians). In (**a**) we compare clinical advice ratings across accuracy, demonstrating that while both groups rated accurate advice as high-quality, only task experts rated inaccurate advice as low-quality. In (**b**) we compare clinical advice ratings across source, demonstrating that only task the experts rated purported human advice as significantly higher quality. There is no significant interaction between advice accuracy and advice source. The boxplots show 25th to 75th percentiles (lower and upper hinges) with the median depicted by the central line; the whiskers extend to a maximum of 1.5× interquartile range (IQR) beyond the boxes. ^*^p ≤ 0.05, ^**^p ≤ 0.001, ns = not significant.

We tested whether the advice quality ratings were affected by the independent variables (see Table 1 for statistics). As expected, participants across the medical specialties correctly rated the quality of the advice on average to be lower if the advice given to them was inaccurate (see Fig. 2a). The effect was much stronger among task experts (i.e., radiologists) than non-experts (i.e., IM/EM physicians). We note that only participants with higher task expertise showed algorithmic aversion by rating the quality of advice to be significantly lower when it came from the AI in comparison to the human. The main effects remained constant when controlled for the inter-individual variables among both physician groups (see Supplementary Table 1). The advice quality rating correlated significantly with the confidence in their diagnosis among both task experts *r*(1102) = 0.43, *p* < 0.001 and non-experts *r*(1014), *p* < 0.001.

**Table 1:** Linear mixed multilevel regression models for advice quality rating.

	Task experts (radiology)				Non-task experts (IM/EM)			
	γ	SE	*t*	*p*	γ	SE	*t*	*p*
Accuracy of the advice	−1.00	0.12	−8.66	<0.001	−0.40	0.12	−3.25	0.002
Source of the advice	0.53	0.15	3.56	0.001	0.22	0.16	1.40	0.165
Accuracy × source	−0.15	0.17	−0.90	0.368	−0.12	0.17	−0.73	0.469

γ = regression coefficient; SE = standard error; *t* = *t*-value; *p* = probability of committing a Type I error, IM = internal medicine, EM = emergency medicine. The regression model also controlled for individual-related variables.

### Diagnostic accuracy is similarly impacted by human/AI advice

**Fig. 3:**
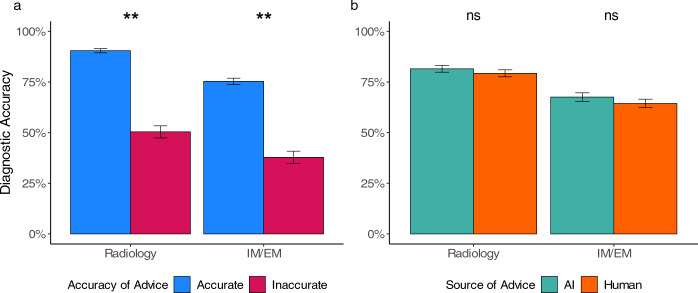
Diagnostic accuracy across advice accuracy and source. We demonstrate the effect of the accuracy of advice and source of advice on diagnostic accuracy for task experts (radiologists) and non-experts (IM/EM physicians). In (**a**) we compare diagnostic accuracy across advice accuracy, demonstrating that both groups perform better when they receive accurate advice. In (**b**) we compare diagnostic accuracy across advice sources, demonstrating that neither group of physicians had a significant difference in diagnostic accuracy depending on the source of advice. There is no significant interaction between advice accuracy and advice source. The error bars represent confidence intervals. ^*^p ≤ 0.05, ^**^p ≤ 0.001, ns = not significant.

We then tested if the participants’ final diagnostic accuracy was affected by the source and/or the accuracy of advice (see Table 2 for statistics). As expected, both participant groups showed a higher diagnostic accuracy when they received accurate advice in comparison to inaccurate advice (see Fig. 3a). Task experts performed 40.10% better and non-experts performed 37.53% better when receiving accurate rather than inaccurate advice. Importantly, the purported source of the advice did not affect participants’ performance (see Fig. 3b). The two main effects and interaction did not change after controlling for the same covariates as above (see Supplementary Table 2). Among the task experts, the two covariates of professional identification (*p* = 0.042) and years of experience (*p* = 0.003) were associated with higher diagnostic accuracy, while none of the covariates affected the diagnostic accuracy among non-experts. Both task experts and non-experts had significantly more confidence in their diagnosis when it was accurate (radiology: *t* = 6.65, *p* < 0.001; IM/EM: *t* = 8.43, *p* < 0.001).

**Table 2:** Logistic mixed multilevel regression models for diagnostic accuracy.

	Task experts (radiology)				Non-task experts (IM/EM)			
	β	SE	*z*	*p*	β	SE	*z*	*p*
Accuracy of the advice	2.39	0.24	9.79	<0.0.001	1.91	0.23	8.26	<0.001
Source of the advice	0.32	0.24	1.33	0.183	0.31	0.18	1.75	0.081
Accuracy × source	−0.29	0.34	−0.85	0.394	−0.51	0.31	−1.62	0.105

β = estimated coefficient; SE = standard error; *z* = *z*-value; *p* = probability of committing a Type I error, IM = internal medicine, EM = emergency medicine. The regression model also controlled for individual-related variables.

### Individual susceptibility and clinical performance varies widely

**Fig. 4:**
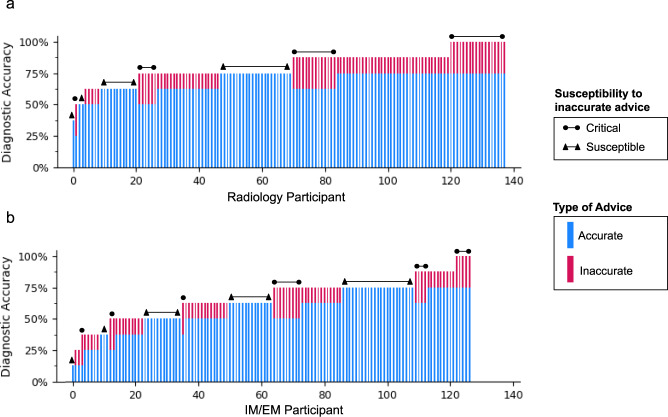
Individual performance. We show the individual performance of radiologists (**a**) and IM/EM physicians (**b**) sorted in increasing order by the number of cases they correctly diagnosed. Each physician’s individual performance is split into cases with accurate advice (the lower, blue part of the bar) and inaccurate advice (the upper, red part of the bar). We further indicate Critical Performers, who always recognize inaccurate advice, and Susceptible Performers, who never do.

As shown in Fig. 4, we investigated the performance of individual radiologists and IM/EM physicians. Radiologists are better performers (13.04% had perfect accuracy, 2.90% had ≤ 50% accuracy), than IM/EM physicians (3.94% had perfect accuracy, 27.56% had ≤ 50% accuracy). We define clinical susceptibility as the propensity to follow incorrect advice, and we find that 41.73% of IM/EM physicians are susceptible, i.e., they always give the wrong diagnosis with inaccurate advice. This is true only for 27.54% of radiologists. Even among physicians with relatively high overall accuracy, a significant portion are susceptible. On the other hand, some physicians are more critical of incorrect advice: 28.26% of radiologists and 17.32% of IM/EM physicians refuted all incorrect advice they were given. Further analysis by advice source is in Supplementary Fig. 1.

### Incorrect advice impacts some clinical cases more than others

**Fig. 5:**
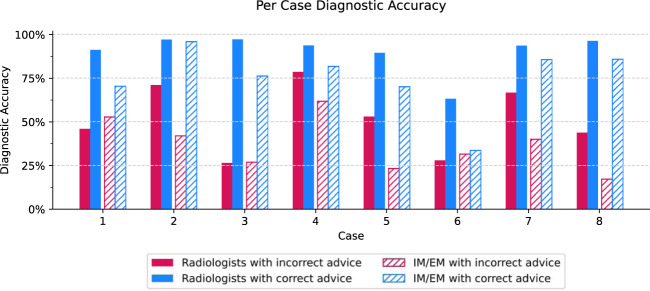
Case performance. Individual case performance amongst radiologists and IM/EM physicians. Each participant reviewed all eight cases; case order and advice accuracy was randomized per participant.

We also looked at participants’ performance on each individual case. As shown in Fig. 5, all cases were impacted by incorrect advice to varying degrees. Case 4 has relatively high average performance under both advice types, for both radiologists and IM/EM physicians. In contrast, Case 6 is more difficult, with generally lower performance under both advice types. Respondents may have misinterpreted the superimposition of ribs as a “pseudo-nodule”^25^; use of the window level/width and magnifying tools in the DICOM viewer should have given the correct diagnoses of a hiatus hernia, apical pneumothorax, and broken rib. Cases that exploit known weaknesses of X-ray evaluators (Cases 3 and 8) had large gaps between diagnostic accuracy under different advice types (inaccurate vs. accurate). For example, in order to correctly diagnose Cases 3 and 8, respondents would need to be aware that pathology is often missed in the retrocardiac window and lung apices^26,27^. There may be a particular risk of over-reliance on inaccurate advice for such cases where physicians fail to recognize known pitfalls in X-ray interpretation or to perform additional analyses to address them.

## Discussion

Hospitals are increasingly interested in implementing AI-enabled clinical support systems to improve clinical outcomes, where an automated system may be viewed as a regulated advice giver^28^. However, how AI-generated advice affects physicians’ diagnostic decision-making in comparison to human-generated advice has been understudied. Our experiments work to build some of this understanding and raise important considerations in the deployment of clinical advice systems.

First, providing diagnostic advice influenced clinical decision-making, whether the advice purportedly came from an AI system or a fellow human. Physicians across expertise levels often failed to dismiss inaccurate advice regardless of its source. In contrast to prior work, we did not find that participants were averse to following algorithmic advice when making their final decision^12–14,29^. We also did not find evidence of algorithmic appreciation, which is in line with previous research exploring behaviors of people with high domain expertise^15,17^. Rather, we found a general tendency for participants to agree with advice; this was particularly true for physicians with less task expertise. The provided diagnosis could have engaged cognitive biases, by anchoring participants to a particular diagnosis, and triggering confirmatory hypothesis testing where participants direct their attention towards features consistent with the advice^30^. Previous research suggests that the anchoring effect and confirmation bias are prevalent in diagnostic decision-making settings, including radiology^31,32^.

This observed over-reliance has important implications for automated advice systems. While physicians are currently able to ask for advice from colleagues, they typically ask for advice after their initial review of the case. Clinical support systems based on AI or more traditional methods could prime physicians to search for confirmatory information in place of conducting a thorough and critical evaluation. If the underlying model has a higher diagnostic accuracy than the physicians using it, patient outcomes may improve overall. However, for high-risk settings like diagnostic decision-making, over-reliance on advice can be dangerous and steps should be taken to minimize it, especially when the advice is inaccurate^33,34^. Prior work suggests that people often discount advice of others relative to their own judgment if they have the option to choose (e.g., see Yaniv and Kleinberger^35^). Therefore, only receiving AI advice upon request may help mitigate the over-reliance problem; further research should investigate the implications of presenting advice automatically versus upon request^36^.

In addition, unlike getting advice from another person, automated recommendations typically do not provide an opportunity for the back-and-forth conversations that characterize many physician interactions, nor involve any notions of uncertainty. Previous research has shown that people prefer advice indicating uncertainty and are more likely to follow sensible advice when provided with notions of confidence^37^. Developing tools that accurately calculate measures of confidence and display them in an understandable way is an important research direction, especially if they may be used by physicians with less task expertise who are at a greater risk to over-trust advice. Indeed, in our study, we found that while physicians often relied on inaccurate advice, they felt less confident about it. Tools that can understandably communicate their own confidence and limitations have the potential to intervene here and prevent over-reliance for these cases where physicians already have some doubt.

Second, we found that physicians with high task expertise rated the quality of the purported AI advice to be worse than purported human advice. Surprisingly, their expressed aversion against algorithmic advice did not affect their reliance on it. Even when controlling for AI- and profession-related individual differences, the effect remained stable. It is possible that the quality rating difference was too small to affect their clinical decision, that the ratings did not reflect their actual attitudes, or that there were other effects such as socially desirable responding^38^. Regardless, this suggests that evaluating the impact of AI systems may be complicated by the discrepancy between physicians’ reported perceptions and their actual behavior.

Finally, we found variability in individual performance across physicians, with some consistently being susceptible to inaccurate advice (including many with relatively high overall accuracy). Further, inaccurate advice was more convincing for certain cases that involved additional interaction or analyses to recognize the error. Previous work surveyed physicians to investigate sources of and ways to reduce practice variability; most physicians agreed that variation should be reduced, and rated having more time to evaluate and apply guidelines most helpful^39^. Decision-aids were also rated as a potentially helpful approach, though our results suggest that they may not reduce variation on their own without further guidelines or training on how to use them. While the online format could have led physicians to review cases less carefully or become more reliant on the advice, we found groups of expert and non-expert participants with perfect performance even with inaccurate advice. Our findings on over-reliance on advice and practice variability relate to known cognitive biases in radiology evaluation^32^. None of the measured covariates significantly predicted susceptibility, for either radiologists or IM/EM physicians. However, investigating what factors do influence susceptibility is an important direction for further research.

The observed variability in physician decisions based on advice also suggests the need for additional guidelines and/or training if decision-aids are deployed. This may take the form of a human-AI calibration phase, where a library of curated test cases is used to demonstrate the system’s strengths and weaknesses, or an AI primer during on-boarding^40^. What this might look like in practice, however, remains an open research question and rich design space.

We also note that there are limitations of the present study. We only tested eight cases in order to keep the study short enough that a sufficient number of physicians would be willing to finish it. While a cross-institutional panel of radiologists chose these cases to be representative of different difficulty levels and pathologies, there is room for expanding upon them and studying behavior with regard to a wider breadth of cases. The study was conducted with a web-based tool, which is inherently limited in its ability to capture decision-making risk and could have impacted clinical behaviors in a different way than a real deployment. It is also difficult to recreate physician conversations regarding patients, which are dynamic and typically characterized by a back-and-forth discussion where clinical information, management advice, and diagnoses are exchanged. While we control for years of experience in our regression models, our sample is skewed towards less experienced physicians, who may be more prone to rely on advice. However, precisely because they may be more susceptible to inaccurate advice^41^, it is important to understand how these less experienced physicians will interact with new technologies and to ensure that potential interventions address their needs effectively.

Overall, the fact that physicians were not able to effectively filter inaccurate advice raises both concerns and opportunities for AI-based decision-support systems in clinical settings. While we are not able to regulate the advice that physicians might give one another, we can aim to design AI systems and interfaces to enable more optimal collaboration.

## Methods

### Participants

The participants were physicians with different task expertise: radiologists were the high-expertise group and physicians trained in internal/emergency medicine (IM/EM) were chosen as the lower expertise group (because they often review chest X-rays but have less experience and training than radiologists). Participants were recruited through emailing staff and residents at hospitals in the US and Canada. The sample consisted of 138 radiologists and 127 IM/EM physicians (see Table 3 for demographics, and Supplementary Table 3 for a more detailed breakdown). The study was exempt from a full ethical review by COUHES, the Institutional Review Board (IRB) for the Massachusetts Institute of Technology (MIT) because the research activities met the criteria for exemption as defined by Federal regulation 45 CFR 46. The experiment complied with all relevant ethical regulations and standards required by COUHES and the Ethical Principles of Psychologists and Code of Conduct outlined by the American Psychology Association (APA). Informed consent was obtained from all participants.

**Table 3:** Participant demographics.

	Task experts	Non-task experts		Total
	Radiology	IM	EM	
*n*	138	80	47	264
Gender* in %				
Female	29.71	36.25	27.66	31.32
Male	69.57	63.75	68.09	67.55
NA	0.72	0.00	4.26	1.13
Years of experience	7.18 (8.12)	3.8 (4.83)	6.03 (8.41)	5.96 (7.47)
Age in %				
18–24	0.72	1.25	0.00	0.75
25–34	68.12	78.75	72.34	72.08
35–44	19.57	15.00	8.51	16.23
45–54	6.52	3.75	10.64	6.42
55–64	2.90	1.25	4.26	2.64
65–74	2.17	0.00	0.00	1.13
NA	0.00	0.00	4.26	0.75
Ethnicity* in %				
White	57.04	52.50	77.08	59.26
Black or African American	1.41	3.75	0.00	1.85
American Indian or Alaska Native	0.70	1.25	0.00	0.74
Asian	30.28	32.50	10.42	27.41
Native Hawaiian or Pacific Islander	0.00	0.00	2.08	0.37
other	2.11	7.50	2.08	3.70
NA	8.45	2.50	8.33	6.67

IM = internal medicine; EM = emergency medicine; *n* = numbers of participants; NA = participants preferred not to answer; years of experience: mean (standard deviation).

*The participants’ gender and ethnicity distribution in each field closely follows the expected distributions according to data from the American Medical Association’s (AMA) Masterfile^42^.

### Data source and case selection

We selected eight cases, each with a chest X-ray (frontal +/− lateral projections), from the open source MIMIC Chest X-ray database^20^. Explicit approval was obtained from the Laboratory for Computational Physiology (LCP) to use the images in our study. A set of candidate cases were chosen by a panel of three radiologists after multiple reviews. The eight final X-rays, clinical histories, radiologic findings, and proposed diagnoses were chosen in collaboration with a senior radiologist. The X-ray IDs and corresponding findings and diagnoses are provided in Supplementary Table 4. The original images can be found via these IDs in the MIMIC-CXR dataset v2.0.0. Six additional radiologists with different levels of experience were consulted to ensure that the accurate and inaccurate advice was plausible and that cases were appropriate for evaluation by physicians with varying levels of expertise. The clinical cases used in this experiment were chosen to reflect clinical practice and included cases designed to test known weaknesses in chest X-ray evaluation^25–27^ (see Supplementary Note 3).

### Experimental design

We conducted a pre-registered web-based experiment (Qualtrics, Provo, UT) where participants saw the chest X-rays (viewable in a fully functional external DICOM viewer), a short clinical vignette, and diagnostic advice that could be used for their final decisions. Participants were asked to (1) evaluate the quality of the advice through a series of questions, and (2) make a final diagnosis (see Fig. 1).

The experiment followed a 2 × 2 mixed factorial design to test the impact of the source of the advice (AI vs. human) as the between-subject factor, and accuracy of the advice (accurate vs. inaccurate) as the within-subject factor. We use a factorial design to examine treatment variations in a single study, where there are two treatments examined and two settings for each treatment (hence 2 × 2). Participants were assigned to one of the two sources of advice for the entire experiment, since receiving advice from both sources may have triggered the participants to purposefully adjust their quality evaluation based on prior attitudes towards AI technology. Participants were randomly assigned six cases with accurate advice and two cases with inaccurate advice. Fewer inaccurate than accurate cases were presented since we felt that any system that was actually deployed would have this property. For the inaccurate cases, a set of plausible radiologic findings were provided to participants; these were designed to support the proposed inaccurate diagnosis if accepted as correct. The distribution of individual-related covariates did not differ significantly for participants in either source of advice groups for both task experts and non-experts (see Supplementary Table 5).

#### Detailed instructions

After entering the online experiment, participants received basic study information and were informed that their participation was entirely voluntary, anonymous, and that they could quit the survey at any time without any adverse consequences. They were informed that the study should take about 10–15 min, and that they would be included in a raffle as compensation for their participation. They were then asked to consent to participate in the study. Prior to starting the experiment, participants were asked to confirm that they were currently practicing physicians (residency included) in the USA or Canada and to select their medical field (radiology, rnternal medicine, or emergency medicine). If they answered that they were not a physician or selected the “other” option for the medical field, the study was terminated.

Participating physicians were then informed that they would be presented with eight patient cases for which they should make a diagnosis. The physicians were told that besides each patient’s clinical history and chest X-rays, they would be given advice in the form of findings and primary diagnoses generated by a particular source. The source was described as either an AI-based model (CHEST-AI) or an experienced radiologist (Dr. S. Johnson). The exact manipulation wordings were:

- AI: “The findings and primary diagnoses were generated by *CHEST-AI*, a well-trained, deep-learning-based artificial intelligence (AI) model with a performance record (regarding diagnostic sensitivity and specificity) on par with experts in the field”.
- Human: “The findings and primary diagnoses were generated by Dr. S. Johnson, an experienced radiologist with a performance record (regarding diagnostic sensitivity and specificity) on par with experts in the field”.

Participants were also asked to do their best to be as accurate as possible. Before proceeding to the actual task, participants learned that they would be presented with static chest X-ray images within the survey but that they should view the chest X-ray images using a DICOM viewer (https://www.pacsbin.com; additional information regarding the DICOM viewer can be found in Supplementary Note 1). The DICOM viewer allowed them to zoom, window, change levels, and look at annotations, among other standard features. The participants first received one example image and were asked to familiarize themselves with the online DICOM viewer. Participants then had to affirm that they had done so and were ready to review their first case. Cases were presented as seen in Fig. 1. Information about individual cases can be found in Supplementary Table 4.

Below the patient case, participants were asked to respond to six questions related to the specific case. These questions were used to measure our two dependent variables (1) advice quality ratings and (2) diagnosis accuracy. See the following subsection for how the variables were combined to calculate the dependent variables. After finishing all eight cases, participants were asked to complete a short survey, including demographics and other variables considered important to control for (individual-related measures).

### Measures

#### Advice quality rating

Each subject indicated their level of agreement (“How much do you agree with the findings?”), usefulness (“How useful are the findings to you for making a diagnosis?”), trustworthiness (“How much do you trust [source of advice]?” and whether they would consult the source of the advice in the future (“Would you consult [source of advice] in the future?”) on 7-point Likert scales from 1 (not at all) to 7 (extremely/definitely). The internal consistency of the advice quality rating was found to be reliable (Cronbach’s α ≥ 0.89). The approach for the advice evaluation was loosely based on a series of studies conducted by Gaertig and Simmons^37^.

#### Diagnostic accuracy

Participants were also asked to provide their final primary diagnosis for each patient case. To do this, the participating physicians were asked if they agreed with the given primary diagnosis (“Do you agree with [primary diagnosis] as the primary diagnosis”). Participants could completely agree with the advised primary diagnosis (“Yes, I agree with this diagnosis”.), agree with it but with slight modification (“Yes, I agree with this diagnosis but would like to add a slight modification”) or disagree with the advice and give an alternative diagnosis (“No, I don’t agree with this diagnosis” followed by “Please provide an alternative primary diagnosis”). We calculated participants’ diagnostic accuracy by taking the accuracy of the provided advice into account:

- If a participant received an accurate diagnosis, both completely agreeing with the advised primary diagnosis as well as agreeing with the advised primary diagnosis, but adding modification was coded as a correct final diagnosis (value = 1). Disagreement with the advised diagnosis was coded as an incorrect final diagnosis (value = 0).
- If a participant received an inaccurate diagnosis, both completely agreeing with the advised primary diagnosis as well as agreeing with the advised primary diagnosis, but adding modification was coded as an incorrect final diagnosis (value = 0). Disagreement with the advised diagnosis was coded as a correct final diagnosis (value = 1).

Each participant’s diagnostic accuracy was calculated by adding the values of all eight cases, dividing by eight, and multiplying the value by 100 to get a percent value. The physicians were also asked to rate their confidence in their diagnosis (“How confident are you with your primary diagnosis?”) from 1 (not at all) to 7 (extremely) for each case. Details for other individual-related measures are in Supplementary Note 2.

### Analysis

As pre-registered (https://osf.io/rx6t8 and https://osf.io/g2njt), the data were analyzed separately for the task experts and non-experts. Two mixed-effects models were calculated for the two dependent variables: (1) quality rating of the advice and (2) accuracy of the final diagnoses. Both dependent variables were regressed on the accuracy of the advice (accurate vs. inaccurate), the source of the advice (AI vs. human), and the interaction between accuracy and source. In regression modeling, we controlled for several individual-related variables: perception of professional identification, beliefs about professional autonomy, self-reported knowledge about and attitude towards AI technology, gender, and years of practice. A linear regression model was used for the quality rating, while a logistic regression model was applied to the accuracy measure. All models included fixed effects for both independent variables and a random effect for the accuracy of the advice variable by participant to account for the non-independence of observations.

### Reporting summary

Further information on research design is available in the Nature Research Reporting Summary linked to this article.

## Supplementary information

Supplementary Information

Reporting Summary

## Data Availability

To maximize the reproducibility of this research, we have uploaded all our analyses and data files to OSF: https://osf.io/rjfqx/. These files allow independent researchers to reconstruct our analysis.

## Code availability

The free programming languages R (3.6.3) and Python (3.7.3) were used to perform all statistical analyses and to create all plots. All code scripts, which include the deployed program packages, are publicly available: https://osf.io/rjfqx/.
