# Supplementary material for: Do as AI say: susceptibility in deployment of clinical decision-aids
Source: NPJ Digit Med. 2021 Feb 19;4:31. doi: 10.1038/s41746-021-00385-9 (PMC7896064; doi:10.1038/s41746-021-00385-9)
Supplement: Supplementary file 1 — Supplementary Information [file 41746_2021_385_MOESM1_ESM.pdf]

Supplementary Materials for

Do as AI Say: Susceptibility in Deployment of Clinical Decision-Aids

Authors: Susanne Gaube[1,2]*†, Harini Suresh[3]*†, Martina Raue[2], Alexander Merritt[4], Seth J. Berkowitz[5], Eva Lermer[6,7], Joseph F. Coughlin[2], John V. Guttag[3], Errol Colak[8,9], Marzyeh Ghassemi[10,11]

Correspondence to: susanne.gaube@ur.de (S.G.) and hsuresh@mit.edu (H.S.); †These authors contributed equally to this work, and should be considered co-first

Supplementary Note 1.

Detailed Statistics

Group Differences: Prior to the main statistical analysis, we checked whether the distribution of participants allocated to the different *source of advice* conditions (AI vs. human advice) differed on any of the individual-related variables (professional identification, belief in professional autonomy, self-reported AI-knowledge, attitude toward AI, years of experience, and gender) which were included as covariates in the subsequent regression models. As Supplementary Table 5 shows, the mean values on all covariate variables did not differ significantly for participants in the two *source of advice* groups among both task experts and non-experts.

Advice Quality Ratings: As reported in the main article, we tested whether the advice quality ratings were affected by the independent variables. There was a significant main effect of the accuracy of advice (accurate vs. inaccurate) on the advice quality rating both among task experts $\chi2(1) = 110.42$, $p < 0.001$ and non-experts $\chi2(1) = 26.38$, $p < 0.001$ (see Manuscript Fig. 2a). There was a significant main effect of the source of the advice (AI vs. human) among the task experts $\chi2(1) = 11.47$, $p < 0.001$ but not among the non-experts $\chi2(1) = 1.51$, $p = 0.219$ (see Manuscript Fig. 2b). There was not a significant interaction between the accuracy of advice and the source of the advice among task experts $\chi2(1) = 0.82$, $p = 0.367$ or non-experts $\chi2(1) = 0.53$, $p = 0.467$. To test if the main effects were affected by the individual-related variables stated above, we included them as covariates in the regression model. We tested if the main effects were affected by the following individual-related variables: professional identification, belief in professional autonomy, self-reported AI-knowledge, attitude toward AI, years of experience, and gender. To do this, we included these six variables as covariates in the regression model. The main effects remained constant when controlled for the inter-individual variables (see Supplementary Table 1) among both physician groups. Including the covariates improved the model fit significantly $p < 0.001$ among the task experts. Higher levels of professional identification, belief in professional autonomy, self-reported AI knowledge, and attitude towards AI among the radiologists resulted in significantly higher quality ratings of the advice. However, among the non-experts, including the covariates did not improve the model fit significantly ($p = 0.088$). Only gender had a significant influence on the quality rating of the advice among internal and emergency physicians, with men giving higher advice quality ratings.

Diagnosis Accuracy: As reported in the main article, we then tested if the correctness of participants' final diagnoses were affected by the source or accuracy of advice. There was a significant main effect of the accuracy of advice both among task experts $\chi2(1) = 187.40$, $p < 0.001$ and non-experts $\chi2(1) = 115.94$, $p < 0.001$ (see Manuscript Fig. 3a). In neither group did the source of the advice affect participant's performance: task experts: $\chi2\ (1) = 1.07$, $p = 0.301$; non-experts: $\chi2(1) = 1.12$, $p = 0.289$ (see Manuscript Fig. 3b). Again, there was neither a significant interaction between the accuracy of advice and the source of the advice among the task experts $\chi2(1) = 0.728$, $p = 0.393$ nor the non-experts $\chi2(1) = 2.63$, $p = 0.105$. All the main effects did not change after controlling for the same covariates as above (see Supplementary Table 2). Including the covariates improved the model fit significantly ($p = 0.001$) among the task experts. Higher levels of professional identification and more work experience among the radiologists resulted in higher diagnostic accuracy. Among the non-experts, including the covariates did not improve the model fit significantly ($p = 0.614$), and no covariate was significantly associated with diagnostic accuracy.

Supplementary Note 2.

Additional Description of Methods

Recruitment Process and Response Rate

During recruitment, we sent emails to residency directors/coordinators at most institutes in the US and Canada that had residency programs in radiology (183 institutions), IM (479) and EM (238), and asked directors/coordinators to forward the email to residents and staff in that field. In addition, when we found physician emails available on the institution's website, we sent recruitment emails to them directly. This process led to approximately 1850 emails sent, resulting in 425 people opening the link to the Qualtrics survey page. 361 people then met our inclusion criteria, consented to participate, and started to look at cases. Finally, 265 people finished looking at cases and answered all of the post-survey questions about demographics, attitudes, and professional identity/autonomy - these are our final participants included in the analysis. This is about a 14.3% response rate given our initial 1850 recruitment emails.

Pre-registered Study Protocols

The pre-registered study protocols (https://osf.io/rx6t8 and https://osf.io/g2njt) can be found on the OSF-project page (https://osf.io/rjfqx/). We report one deviation: while we planned to recruit 128 radiologists and 128 IM/EM physicians, we ultimately obtained 138 and 127 participants respectively.

DICOM viewer

Participants were able to see the chest x-rays in the Digital Imaging and Communications in Medicine (DICOM) format, which is commonly used for radiologic images, and allows grouping multiple images and metadata together in a lossless format. The DICOMS were accessible in a fully functional external DICOM viewer called Pacsbin "Pacsbin is a platform developed by radiologists as an attempt to make HIPAA compliant radiology teaching cases easier to create, view, and share. Purpose: The primary goal of Pacsbin is to bring a fully-featured PACS environment to the web for teaching cases and research. Modern web technologies, in particular Javascript, HTML5, and CSS3 have enabled the creation of fast, highly functional radiology imaging programs in the browser. Pacsbin leverages the fantastic open source Cornerstone library from Chris Hafey, adding education specific tools, an anonymization pathway, and a way to organize your cases and save them forever. Features: Full DICOM images, with support for the most common image manipulation tools. Editing interface for creators of case content, including annotations and case notes. Create links from case notes to specific images and window/level settings, allowing a guided tour through pertinent imaging findings. Automated anonymization pipeline, using DICOM standard anonymization techniques. Upload images directly, or link to an enterprise PACS for single click case creation." (Information from https://www.pacsbin.com/docs/about)

Additional Measures

- **Professional identification**: A five-item scale (e.g., "In general, when someone praises doctors, it feels like a personal compliment."[1]) answered on a 7-point Likert scale from 1 (*strongly disagree*) to 7 (*strongly agree*); Cronbach's α = 0.77.

[1] D. R. Hekman, H. K. Steensma, G. A. Bigley, J. F. Hereford, Effects of organizational and professional identification on the relationship between administrators' social influence and professional employees' adoption of new work behavior. *J. Appl. Psychol.* **94**, 1325–1335 (2009).

- **Belief in professional autonomy:** A four-item scale (e.g., "Individual physicians should make their own decisions in regard to what is to be done in their work."[2]) answered on a 7-point Likert scale from 1 (*strongly disagree*) to 7 (*strongly agree*); Cronbach's $\alpha = 0.68$.
- **Self-reported AI knowledge:** "How would you consider your own general knowledge of artificial intelligence (AI)? (*I have no knowledge, Novice: I have heard of AI, Intermediate: I have read media articles or have listened to news about AI technologies, Advanced: I have used AI-based tools and have some understanding of how they work, Expert: For example, I am an academic or industry researcher in AI*).
- **Attitude toward AI:** Three item scale ("How much do you agree with the following statements? - AI will make most people's lives better."; "How much do you agree with the following statements? - AI is dangerous to society."; "How much do you agree with the following statements? - AI poses a threat to my career.") answered on a 7-point Likert scale from 1 (*strongly disagree*) to 7 (*strongly agree*); Cronbach's $\alpha = 0.57$.
- **Years of experience:** "How many years of experience do you have as a physician (starting from your first year of residency)?" (*open answer format*).
- **Gender:** "What is your gender?" (*male, female, other, prefer not to answer*).
- **Age**: "What is your age?"; single choice answer format (*18-24, 25-34, 35-44, 45-54, 55-64, 65-74, 75-84, 85 or older, prefer not to answer*).
- **Ethnicity:** "What is your ethnicity?" (*White (Europe, Middle East, North Africa), Black or African American (Africa), American Indian or Alaska Native (North America, South America, Central America), Asian (Far East, Southeast Asia, Indian), Native Hawaiian or Pacific Islander (Hawaii, Guam, Samoa, Pacific Islands), other, prefer not to answer*).

Supplementary Note 3.

Patient Case Information

- **Case 1** is a normal chest x-ray. The potential pitfall is misinterpreting the left breast shadow as pneumonia.
- **Case 2** is an uncommon fracture-dislocation injury. A systematic search pattern in interpreting chest x-rays should include the sterno-clavicular joint. Less experienced physicians may be unaware of this injury and misinterpreted the fracture fragment as a pleural plaque which is a more commonly encountered finding.
- **Case 3** illustrates the importance of adjusting window levels and width when assessing the retrocardiac space. Normally the retrocardiac region contains traversing pulmonary vessels that taper peripherally. Careful scrutiny of case 3 reveals an air and soft tissue density mass in the retrocardiac region. This finding is characteristic of a hiatal hernia, a benign but potentially symptomatic entity. The cardiac silhouette on frontal chest radiographs can decrease conspicuity of important pathology that can occur in the retrocardiac space such as lung cancers, enlarged lymph nodes, and aortic aneurysms.
- In **Case 4** attentive interrogation of the right lung apex should have led individuals to recognize a visceral pleural edge with no distal lung markers which are characteristic findings of a pneumothorax. It has been well established that pathology at the lung apices may be missed due to the many overlapping anatomical structures in this region (25,26).

[2] T. J. Hoff, Professional commitment among US physician executives in managed care. *Soc. Sci. Med.* **50**, 1433-1444 (2000).

- **Case 5** requires respondents to recognize an ill-defined right upper lung opacity. This radiographic finding and clinical history of cough should lead to the correct diagnosis of pneumonia. Respondents may have misinterpreted the ill-defined opacity as vascular markers or the superimposition of anatomical structures such as ribs.
- In **Case 6**, a focal area of increased density appears to project over the right upper lung. A first instinct may be to consider this a pulmonary nodule (which was provided as incorrect advice in the experiment). However, upon closer review the area of increased density can be accounted for by overlapping of the third anterior and sixth posterior ribs. The correct diagnosis of an acute rib fracture can be made by identifying the step deformity of the third anterior right rib. The superimposition of anatomical structures is a well-documented cause of "pseudo-nodules" (27).
- **Case 7** requires respondents to integrate the clinical history and multiple radiograph findings to arrive at the correct diagnosis of pulmonary edema.
- In **Case 8**, a less vigilant or experienced respondent may have misinterpreted the visceral pleural edge as a rib. It has been well established that pathology at the lung apices may be missed due to the many overlapping anatomical structures in this region (25,26). Attentive interrogation of the left lung apex in case 8 should have led individuals to recognize a visceral pleural edge with no distal lung markers which are characteristic findings of a pneumothorax.

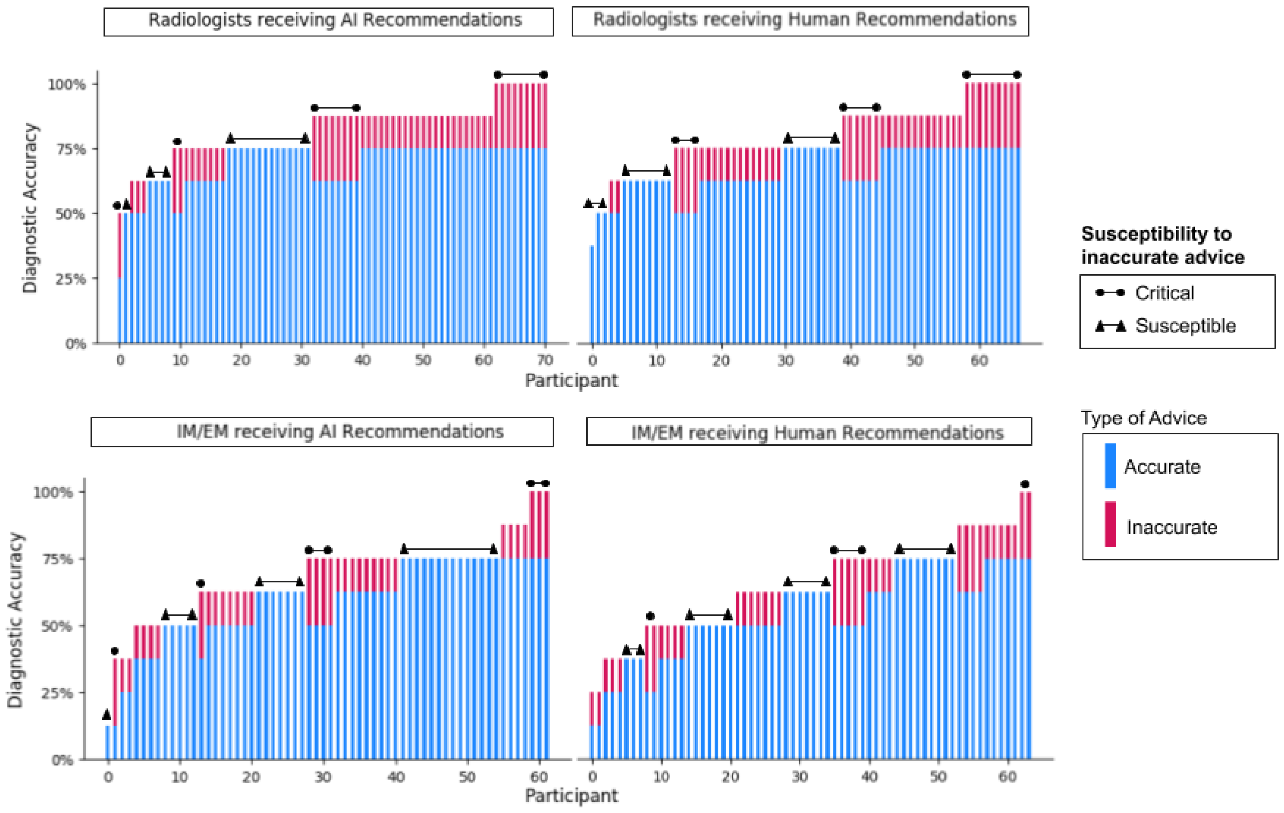

Supplementary Figure 1.

We show the individual performance of radiologists and IM/EM physicians sorted in increasing order by the number of cases they correctly diagnosed, and split up by whether they received advice labeled as coming from an AI or human source. Each physician's individual performance is split up by their performance on cases with accurate advice (the lower, blue part of the bar) and inaccurate advice (the upper, red part of the bar). We further indicate **Critical Performers**, who always recognize inaccurate advice, and **Susceptible Performers**, who never do. While the distribution of performance levels is quite different across expertise groups, it does not differ much by the source of advice within an expertise group.

Supplementary Table 1.
Linear mixed multilevel regression models for advice quality ratings

	Task experts (radiology)				Non-task experts (IM/EM)			
Model 1	γ	*SE*	*t*	*p*	γ	*SE*	*t*	*p*
Intercept	5.03	0.10	48.46	<0.001	4.85	0.11	43.40	<0.001
Accuracy of the advice	-1.00	0.12	-8.66	<0.001	-0.40	0.12	-3.25	0.002
Source of the advice	0.53	0.15	3.56	0.001	0.22	0.16	1.40	0.165
Accuracy x Source	-0.15	0.17	-0.90	0.368	-0.12	0.17	-0.73	0.469
Model 2 (incl. covariates)								
Intercept	1.74	0.56	3.08	0.002	5.06	0.67	7.55	<0.001
Accuracy of the advice	-1.00	0.11	-8.98	<0.001	-0.40	0.12	-3.25	0.001
Source of the advice	0.55	0.13	4.17	<0.001	0.23	0.15	1.48	0.141
Accuracy x Source	-0.15	0.16	-0.94	0.351	-0.12	0.17	-0.73	0.469
Identification	0.12	0.06	2.05	0.043	0.04	0.07	0.61	0.545
Autonomy	0.27	0.07	4.00	<0.001	0.03	0.07	0.46	0.646
AI Knowledge	0.21	0.09	2.33	0.021	-0.06	0.10	-0.61	0.542
AI Attitude	0.25	0.06	4.27	<0.001	0.04	0.08	0.47	0.636
Experience	0.01	0.01	1.64	0.104	0.00	0.01	-0.19	0.849
Gender	-0.20	0.12	-1.65	0.102	-0.40	0.13	-3.11	0.002

Note. γ = regression coefficient; *SE* = standard error; *t* = t-value; *p* = probability of committing a Type I error, IM = internal medicine, EM = emergency medicine.

Supplementary Table 2.

Logistic mixed multilevel regression models for diagnostic accuracy

	Task experts (radiology)				Non-task experts (IM/EM)			
Model 1	β	*SE*	*z*	*p*	β	*SE*	*z*	*p*
Intercept	-2.41	0.18	-13.68	<0.001	-1.30	0.13	-9.71	<0.001
Accuracy of the advice	2.39	0.24	9.79	<0.001	1.91	0.23	8.26	<0.001
Source of the advice	0.32	0.24	1.33	0.183	0.31	0.18	1.75	0.081
Accuracy x Source	-0.29	0.34	-0.85	0.394	-0.51	0.31	-1.62	0.105
Model 2 (incl. covariates)								
Intercept	-0.17	0.81	-0.21	0.835	-1.20	0.70	-1.71	0.087
Accuracy of the advice	2.43	0.25	9.86	<0.001	1.91	0.23	8.26	<0.001
Source of the advice	0.30	0.24	1.23	0.217	0.29	0.18	1.63	0.104
Accuracy x Source	-0.27	0.34	-0.78	0.436	-0.50	0.31	-1.61	0.108
Identification	-0.15	0.08	-2.03	0.042	-0.02	0.07	-0.28	0.780
Autonomy	-0.09	0.10	-0.95	0.342	0.01	0.08	0.08	0.937
AI Knowledge	-0.20	0.13	-1.55	0.120	-0.08	0.10	-0.76	0.445
AI Attitude	-0.07	0.08	-0.87	0.383	-0.02	0.08	-0.29	0.771
Experience	-0.04	0.01	-2.97	0.003	0.00	0.01	0.00	0.996
Gender	0.01	0.18	0.06	0.950	0.23	0.13	1.74	0.082

Note. β = estimated coefficient; *SE* = standard error; *z* = z-value; *p* = probability of committing a Type I error, IM = internal medicine, EM = emergency medicine.

Supplementary Table 3.
Descriptive statistics on the additional measures, *M* (*SD*) or % of participants

	Task experts (radiology)	Non-task experts (IM/EM)	Total
Professional identification	4.65 (0.94)	4.66 (1.11)	4.65 (1.02)
Belief in professional autonomy:	3.96 (0.92)	3.81 (1.01)	3.89 (0.96)
Self-reported AI knowledge:			
No knowledge	0.00%	3.94%	1.89%
Novice	15.33%	30.71%	22.73%
Intermediate	64.23%	51.18%	57.95%
Advanced	18.98%	14.17%	16.67%
Expert	1.46%	0.00%	0.76%
Attitude toward AI:	4.77 (1.05)	4.89 (0.96)	4.83 (1.01)
Gender in %			
Female	29.71%	33.07%	31.32%
Male	69.57%	65.35%	67.55%
NA	0.72%	1.57%	1.13%
Years of Experience	7.18 (8.12)	4.63 (6.46)	5.96 (7.47)
Age in %			
18-24	0.72%	0.79 %	0.75 %
25-34	68.12%	76.38 %	72.08 %
35-44	19.57%	12.60 %	16.23 %
45-54	6.52%	6.30 %	6.42 %
55-64	2.90%	2.36 %	2.64 %
65-74	2.17%	0.00 %	1.13 %
NA	0.00%	1.57 %	0.75 %
Ethnicity in %			
White	57.04%	61.72%	59.26%
Black or African American	1.41%	2.34%	1.85%
American Indian or Alaska Native	0.70%	0.78%	0.74%
Asian	30.28%	24.22%	27.41%
Native Hawaiian or Pacific Islander	0.00%	0.78%	0.37%
other	2.11%	5.47%	3.70%
NA	8.45%	4.69%	6.67%

Note. N = 264, $n_{(Radiology)}$ = 138, $n_{(\mathrm{IM/EM})}$ = 127, IM = internal medicine, EM = emergency medicine, NA = participants preferred not to answer, AI = artificial intelligence

Supplementary Table 4.
Patient case information

Case # and ID	Link to Pacsbin	MIMIC ID	Patient information	Findings		Diagnosis	
				Accurate	Inaccurate	Accurate	Inaccurate
#1 PT001	P1	p1088357 2\s01	A 26-year-old female presenting to the Emergency Department with chest pain.	• Normal heart size<br>• No airspace opacification<br>• No pleural effusion<br>• No pneumothorax	• Normal heart size<br>• Lingular airspace opacification<br>• No pleural effusion<br>• No pneumothorax	Normal	Lingular pneumonia
#2 PT002	P2	p1016555 5\s04	A 51-year-old male presenting to his Primary Care Physician with chronic chest pain.	• Normal heart size<br>• No airspace opacification<br>• No pleural effusion<br>• No pneumothorax<br>• Dislocated right sternoclavicular joint	• Normal heart size<br>• No airspace opacification<br>• No pleural effusion<br>• No pneumothorax<br>• Peripherally calcified nodule at the right apex	Right sterno-clavicular dislocation	Right pleural plaque
#3 PT005	P3	p1045416 5\s01	A 32-year-old female with chronic cough.	• Normal heart size<br>• Retrocardiac opacity<br>• No pleural effusion<br>• No pneumothorax<br>• Fourth right rib osteotomy	• Normal heart size<br>• No airspace opacification<br>• No pleural effusion<br>• No pneumothorax<br>• Fourth right rib osteotomy	Hiatus hernia	Normal
#4 PT007	P4	p1045416 5\s01	A 57-year-old male with shortness of breath.	• Mild cardiomegaly<br>• Visceral pleural edge at right apex<br>• Right basilar atelectasis<br>• Small right pleural effusion<br>• Right rib fractures	• Normal heart size<br>• Right lower lobe airspace opacification<br>• Small right pleural effusion<br>• Right rib fractures	Right pneumo-thorax	Right lower lobe pneumonia

Case # and ID	Link to Pacsbin1	MIMIC ID	Patient information	Findings		Diagnosis	
				Accurate	Inaccurate	Accurate	Inaccurate
#5 PT010	P5	p1016567 2\s10	A 63-year-old male presenting to the Emergency Department with cough.	• Normal heart size<br>• Right upper lobe airspace opacification<br>• Small pleural effusion<br>• No pneumothorax	• Normal heart size<br>• Peribronchial cuffing<br>• Kerley B lines<br>• Small pleural effusion<br>• No pneumothorax	Right upper lobe pneumonia	Pulmonary venous congestion
#6 PT011	P6	p1017611 8\s01	A 46-year-old male with a 10-pack year history of smoking.	• Normal heart size<br>• Focal opacity projecting over right upper lung<br>• No pleural effusion<br>• No pneumothorax	• Normal heart size<br>• Focal opacity projecting over right upper lung<br>• No pleural effusion<br>• No pneumothorax	Rib fracture	Pulmonary nodule
#7 PT014	P7	p1015267 5\s05	A 64-year-old male with shortness of breath	• Moderate cardiomegaly<br>• Mild vascular redistribution<br>• Interstitial thickening<br>• Peribronchial cuffing<br>• Small bilateral pleural effusions<br>• Basilar atelectasis	• Moderate cardiomegaly<br>• Left basilar air space opacification<br>• Small bilateral pleural effusions	Pulmonary edema	Left lower lobe pneumonia
#8 PT015	P8	p1042665 0\s01	A 19-year-old male presenting to the Emergency Department with chest pain.	• No cardiomegaly<br>• Visceral pleural edge at left apex<br>• Small left pleural effusion<br>• Left basilar atelectasis	• No cardiomegaly<br>• Left basilar air opacification<br>• Small left pleural effusion	Left pneumo-thorax	Left lower lobe pneumonia

Note. The values in the “Link to Pacsbin” column are links to view the x-rays in a web-based DICOM viewer. The x-rays are better viewed in this way, since they lose quality when converted to JPG files and made smaller to fit on a page.

Supplementary Table 5.

Sample covariates using t-test for equality of means

	Task experts (radiology)			Non-task experts (IM/EM)		
Covariates	*df*	*t*	*p*	*df*	*t*	*p*
Identification	133.95	-1.20	0.231	124.28	-1.03	0.305
Autonomy	134.63	0.47	0.636	118.51	-0.56	0.579
AI Knowledge	134.78	0.90	0.367	122.95	0.75	0.454
AI Attitude	130.19	-0.09	0.925	125.00	-0.49	0.623
Experience	131.88	-0.52	0.601	123.99	-0.06	0.951
Gender	133.63	0.45	0.651	116.71	-0.75	0.453

Note. *df* = degrees of freedom; *t* = t-value; *p* = probability of committing a Type I error, IM = internal medicine, EM = emergency medicine.
