# Supplementary material for: Do as AI say: susceptibility in deployment of clinical decision-aids
Source: NPJ Digit Med. 2021 Feb 19;4:31. doi: 10.1038/s41746-021-00385-9 (PMC7896064; doi:10.1038/s41746-021-00385-9)
Supplement: Supplementary file 2 — Reporting Summary [file 41746_2021_385_MOESM2_ESM.pdf]

nature research

Corresponding author(s): Susanne Gaube, Harini Suresh

Last updated by author(s): Dec 5, 2020

Reporting Summary

Nature Research wishes to improve the reproducibility of the work that we publish. This form provides structure for consistency and transparency in reporting. For further information on Nature Research policies, see our Editorial Policies and the Editorial Policy Checklist.

Statistics

For all statistical analyses, confirm that the following items are present in the figure legend, table legend, main text, or Methods section.

n/a	Confirmed	
☐	☒	The exact sample size (n) for each experimental group/condition, given as a discrete number and unit of measurement
☐	☒	A statement on whether measurements were taken from distinct samples or whether the same sample was measured repeatedly
☐	☒	The statistical test(s) used AND whether they are one- or two-sided<br>*Only common tests should be described solely by name; describe more complex techniques in the Methods section.*
☐	☒	A description of all covariates tested
☒	☐	A description of any assumptions or corrections, such as tests of normality and adjustment for multiple comparisons
☐	☒	A full description of the statistical parameters including central tendency (e.g. means) or other basic estimates (e.g. regression coefficient) AND variation (e.g. standard deviation) or associated estimates of uncertainty (e.g. confidence intervals)
☐	☒	For null hypothesis testing, the test statistic (e.g. F, t, r) with confidence intervals, effect sizes, degrees of freedom and P value noted<br>*Give P values as exact values whenever suitable.*
☒	☐	For Bayesian analysis, information on the choice of priors and Markov chain Monte Carlo settings
☐	☒	For hierarchical and complex designs, identification of the appropriate level for tests and full reporting of outcomes
☒	☐	Estimates of effect sizes (e.g. Cohen's d, Pearson's r), indicating how they were calculated

Our web collection on statistics for biologists contains articles on many of the points above.

Software and code

Policy information about availability of computer code

Data collection	The data for this paper was collected using Qualtrics software, Version March 2020 of Qualtrics (https://www.qualtrics.com).
Data analysis	Data analysis was conducted using R 3.6.3 and Python 3.7.3.

For manuscripts utilizing custom algorithms or software that are central to the research but not yet described in published literature, software must be made available to editors and reviewers. We strongly encourage code deposition in a community repository (e.g. GitHub). See the Nature Research guidelines for submitting code & software for further information.

Data

Policy information about availability of data

All manuscripts must include a data availability statement. This statement should provide the following information, where applicable:

- Accession codes, unique identifiers, or web links for publicly available datasets
- A list of figures that have associated raw data
- A description of any restrictions on data availability

To maximize the reproducibility of this research, we have uploaded all our analyses and data files to OSF: https://osf.io/rjfqx/. These files allow independent researchers to reconstruct our analysis.

Field-specific reporting

Please select the one below that is the best fit for your research. If you are not sure, read the appropriate sections before making your selection.

☐ Life sciences ☒ Behavioural & social sciences ☐ Ecological, evolutionary & environmental sciences

For a reference copy of the document with all sections, see nature.com/documents/nr-reporting-summary-flat.pdf

Behavioural & social sciences study design

All studies must disclose on these points even when the disclosure is negative.

Study description	In this study, physicians received chest x-rays and diagnostic advice, some of which was inaccurate, and were asked to evaluate advice quality and make diagnoses. All advice was generated by human experts, but some was labeled as coming from an AI system. The study used a quantitative mixed-methods experimental design.
Research sample	The participants were physicians with different task expertise: radiologists (n = 138) were the high expertise group and physicians trained in internal/emergency medicine (IM/EM, n = 127) were chosen as the lower expertise group because they often review chest x-rays but have less experience and training than radiologists. The participants' gender and ethnicity distribution (described along with other demographics in Table 3) in each field closely follows the expected distributions according to data from the American Medical Association's (AMA) Masterfile.
Sampling strategy	According to an a priori power analyses, we aimed to collect responses from 128 task experts and 128 non-experts. This number was calculated expecting a moderate effect size of $f = 0.25$, α-error probability of .05, and power (1 - β-error probability) of .80. Participants were randomly allocated to the two groups (corresponding to different sources of advice).
Data collection	The study was conducted online using Qualtrics. The researchers were not present.
Timing	Data collection occurred from 9/11/2019 to 3/1/2020.
Data exclusions	Data were excluded if the participants did not fall into the criteria for participation (Radiologists or IM/EM physicians in the US or Canada) or did not complete the full survey.
Non-participation	During recruitment, we sent emails to residency directors/coordinators at most institutes in the US and Canada that had residency programs in radiology (183 institutions), IM (479) and EM (238), and asked if directors/coordinators to forward the email to residents and staff in that field. In addition, when we found physician emails available on the institution's website, we sent recruitment emails to them directly. This process led to approximately 1850 emails sent, resulting in 425 people opening the link to the Qualtrics survey page. 361 people then met our inclusion criteria, consented to participate, and started to look at cases. Finally, 265 people finished looking at cases and answered all of the post-survey questions about demographics, attitudes, and professional identity/autonomy - these are our final participants included in the analysis. This is about a 14.3% response rate given our initial 1850 recruitment emails.
Randomization	Participants were randomly allocated to groups, and the order of patient cases was also randomized for each participant.

Reporting for specific materials, systems and methods

We require information from authors about some types of materials, experimental systems and methods used in many studies. Here, indicate whether each material, system or method listed is relevant to your study. If you are not sure if a list item applies to your research, read the appropriate section before selecting a response.

Materials & experimental systems

n/a	Involved in the study	
☒	☐	Antibodies
☒	☐	Eukaryotic cell lines
☒	☐	Palaeontology and archaeology
☒	☐	Animals and other organisms
☐	☒	Human research participants
☒	☐	Clinical data
☒	☐	Dual use research of concern

Methods

n/a	Involved in the study	
☒	☐	ChIP-seq
☒	☐	Flow cytometry
☒	☐	MRI-based neuroimaging

Human research participants

Policy information about studies involving human research participants

Population characteristics	See above.

Recruitment	See above.
Ethics oversight	The study protocol was approved by the MIT Committee of the Use of Humans as Experimental Subjects, and was certified as exempt from IRB review under Category 3 (benign behavioral intervention).

Note that full information on the approval of the study protocol must also be provided in the manuscript.
